# Prevalence of Carbapenem Resistance Genes among *Acinetobacter baumannii* Isolated from a Teaching Hospital in Taiwan

**DOI:** 10.3390/antibiotics12091357

**Published:** 2023-08-23

**Authors:** Pai-Wei Su, Emirlyn Cheng Yang, Sin-Hua Moi, Cheng-Hong Yang, Li-Yeh Chuang

**Affiliations:** 1 General Education Center, Wenzao Ursuline University of Languages, Kaohsiung 80793, Taiwan; 97044@mail.wzu.edu.tw; 2 Department of Post-Baccalaureate Medicine, Kaohsiung Medical University, Kaohsiung 80708, Taiwan; u112000033@gap.kmu.edu.tw; 3 Graduate Institute of Clinical Medicine, Kaohsiung Medical University, Kaohsiung 80708, Taiwan; moish@kmu.edu.tw; 4 Department of Information Management, Tainan University of Technology, Tainan 71002, Taiwan; 5 Ph. D. Program in Biomedical Engineering, Kaohsiung Medical University, Kaohsiung, 80708, Taiwan; 6 Institute of Biotechnology and Chemical Engineering, I-Shou University, Kaohsiung 84001, Taiwan

**Keywords:** *Acinetobacter baumannii*, antibiotic resistance, genotyping

## Abstract

The problem of antibiotic-resistant strains has become a global public issue; antibiotic resistance not only limits the choice of treatments but also increases morbidity, mortality and treatment costs. The multi-drug resistant *Acinetobacter baumannii* is occurring simultaneously in hospitals and has become a major public health issue worldwide. Although many medical units have begun to control the use of antibiotics and paid attention to the issue of drug resistance, understanding the transmission pathways of clinical drug-resistant bacteria and drug-resistant mechanisms can be effective in real-time control and prevent the outbreak of antibiotic-resistant pathogens. In this study, a total of 154 isolates of *Acinetobacter baumannii* obtained from Chia-Yi Christian Hospital in Taiwan were collected for specific resistance genotyping analysis. Ten genes related to drug resistance, including *bla*OXA-51-like, *bla*OXA-23-like, *bla*OXA-58-like, *bla*OXA-24-like, *bla*OXA-143-like, *tnp*A, IS*Aba*1, *bla*PER-1, *bla*NDM and *bla*ADC, and the repetitive element (ERIC2) were selected for genotyping analysis. The results revealed that 135 *A. baumannii* isolates (87.6%) carried the *bla*OXA-51-like gene, 4.5% of the isolates harbored the *bla*OXA-23-like gene, and 3.2% of the isolates carried the *bla*OXA-58-like gene. However, neither the *bla*OXA-24-like nor *bla*OXA-143-like genes were detected in the isolates. Analysis of ESBL-producing strains revealed that *bla*NDM was not found in the test strains, but 38.3% of the test isolates carried *bla*PER-1. In addition, *bla*ADC, *tnp*A and IS*Aba*1genes were found in 64.9%, 74% and 93% of the isolates, respectively. Among the carbapenem-resistant strains of *A. baumannii*, 68% of the isolates presenting a higher antibiotic resistance carried both *tnp*A and IS*Aba*1 genes. Analysis of the relationship between their phenotypes (antibiotic resistant and biofilm formation) and genotypes (antibiotic-resistant genes and biofilm-related genes) studied indicated that the *bap*, *omp*A, IS*Aba*1and *bla*OXA-51 genes influenced biofilm formation and antibiotic resistance patterns based on the statistical results of a hierarchical clustering dendrogram. The analysis of the antibiotic-resistant mechanism provides valuable information for the screening, identification, diagnosis, treatment and control of clinical antibiotic-resistant pathogens, and is an important reference pointer to prevent strains from producing resistance.

## 1. Introduction

*Acinetobacter baumannii*, a gram-negative opportunistic pathogen, thrives within hospital settings, enduring adverse circumstances like dehydration, lack of nutrients, and antimicrobial interventions [1,2]. In the realm of antimicrobial investigation, the identification of numerous antibiotics was sequentially succeeded by the rise of antibiotic resistance. Presently, the escalation of bacterial antibiotic resistance persists. Nonetheless, the scientific community and pharmaceutical industry have encountered challenges in developing novel drugs to substitute the current antimicrobial agents that have succumbed to resistance [3]. Major clinically relevant characteristics of *A. baumanni*i are its high natural resistance to antibiotics and excellent ability to upregulate its innate antimicrobial resistance mechanisms and acquire relevant foreign mechanisms [4]. In recent years, there has been a notable prevalence of infections and outbreaks caused by multidrug-resistant *A. baumannii* (MDRAB), a trend that has been globally documented for at least the past two decades. MDRAB is an emerging pathogen in healthcare settings, particularly ICUs, and displaying resistance to more than two out of the five specified classes of antibiotics: antipseudomonal cephalosporins (ceftazidime or cefepime), antipseudomonal carbapenems (imipenem (IPM) or meropenem), ampicillin/sulbactam, fluoroquinolones (ciprofloxacin or levofloxacin), and aminoglycosides (gentamicin, tobramycin or amikacin) [5,6].

Carbapenems, including IPM or meropenem, are antimicrobial agents effective for treating *A. baumannii* infections [7]. However, resistance to carbapenems, mechanical ventilation, and malignancy are associated with high mortality rates in patients with *A. baumannii* bacteremia [8]. Carbapenem-resistant *A. baumannii*, which can survive for a longer period in hospitals, poses a serious threat to hospitalized patients. *Acinetobacter* species resist carbapenems by producing various carbapenemase enzymes; Class B metallo-β-lactamases (MBLs) and Class D oxacillinases (OXAs) are common [9]. Managing infectious diseases is a major challenge, and MDR is often mediated by genetically mobile elements, such as plasmids, transposons, and integrons [10]. IS*Aba*1 is an insertion sequence (IS) that is widely distributed in *A. baumannii*, and many isolates are located upstream of the carbapenem-resistant oxacillinase genes, such as *bla*OXA-23, *bla*OXA-51, and *bla*OXA-58 [11]. The control of *A. baumannii* infections is seen as achievable through two primary approaches: the creation of novel antibiotics targeting MDRAB and the adoption of infection-control strategies [12].

Drug resistance mechanisms primarily encompass enzymes, membrane proteins, efflux pumps, and beneficial mutations [13]. Research into these underlying mechanisms serves as a fundamental basis for the effective use and development of antibiotics, as well as the exploration of novel treatment strategies. One particular area of significant interest among biomedical scientists is understanding the relationship between multidrug-resistant (MDR) phenotypes and biofilm-forming capacity, along with the correlation between genotypes associated with biofilm formation and antibiotic resistance [14]. These factors play a crucial role in influencing infection outcomes. While certain clinical and epidemiological studies have explored the correlation between virulence, biofilm production, and antibiotic resistance gene associations, and some reports have utilized whole genome sequencing [15], it is important to note that resistance caused by biofilms is specific, and the related genes are not direct factors contributing to *Acinetobacter baumannii* resistance [13]. Therefore, further research is necessary to elucidate the specific mechanisms involved.

In our previous study, we conducted an investigation into the interplay among biofilm-forming capabilities, antibiotic resistance, and biofilm-related genes within 154 *A. baumannii* isolates sourced from a teaching hospital in Taiwan [16]. The outcomes of our experiments revealed that the isolates exhibiting resistance to multiple drugs typically demonstrated elevated biofilm formation. Among the collected strains, the prevalence of biofilm-related genes, namely *bap*, *bla*PER-1, *csu*E and *omp*A, was 79.2%, 38.3%, 91.6%, and 68.8%, respectively. These findings underscored a noteworthy association between antibiotic resistance, biofilm development, and the presence of these associated genes.

In this study, we investigated the 154 clinical isolates for the presence of carbapenemase genes (*bla*OXA-51-like, *bla*OXA-23-like, *bla*OXA-58-like, *bla*OXA-24-like, *bla*OXA-143-like, *bla*PER-1, *bla*NDM and *bla*ADC), IS*Aba*1 and *tnp*A genes. Building upon our previous experimental results, we also analyzed the relationship between their phenotypes (antibiotic resistance and biofilm formation) and genotypes (antibiotic-resistant genes and biofilm-related genes). Monitoring hospital-acquired infections, especially when coupled with an analysis of the phenotypic and genotypic characteristics of prominent MDR pathogens, facilitates the enactment of containment protocols amidst infection outbreaks and the formulation of effective infection control strategies [15].

## 2. Results

### 2.1. Molecular Characterization of the Antimicrobial Resistance Determinants

**Figure 1:**
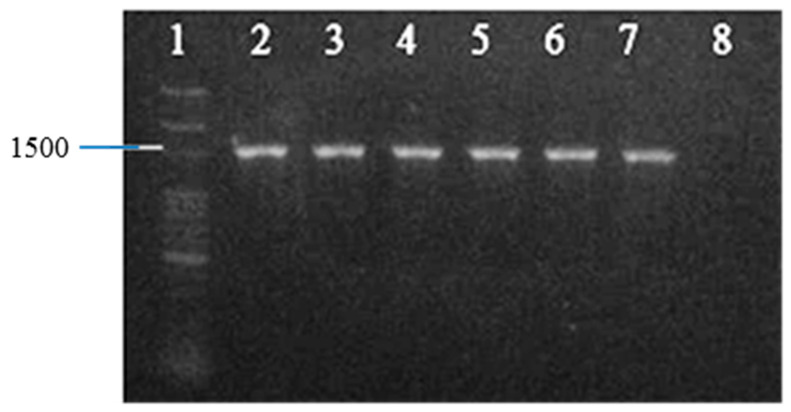
PCR amplification of 16S rRNA gene in *A. baumannii* isolates. Lane 1, 100 bp DNA ladder; Lanes 2–7, positive isolate for 16S rRNA gene; Lane 8: negative control.

The confirmation of *Acinetobacter* species was performed using a Polymerase Chain Reaction (PCR) assay of the 16S rRNA gene (Figure 1). In this study, all isolates were subjected to PCR for the detection of eight beta-lactamase genes (*bla*OXA-51-like, *bla*OXA-23-like, *bla*OXA-58-like, *bla*OXA-24-like, *bla*OXA-143-like, *bla*NDM, *bla*ADC and *bla*PER-1) and *tnp*A as well as IS*Aba*1 elements (Table 1). The results revealed that 135 *A. baumannii* isolates (87.6%) carried the *bla*OXA-51-like gene, 4.5% of the isolates harbored the *bla*OXA-23-like gene, and 3.2% of the isolates carried the *bla*OXA-58-like gene. However, neither the *bla*OXA-24-like nor *bla*OXA-143-like genes were detected in the isolates. The most common insertion sequence (IS) in *A. baumannii*, IS*Aba*1 gene, was found in 88.3% of the isolates. Among the 154 isolates, 100 carbapenem-resistant isolates (the ceftazidime and imipenem-resistant strains) and 54 carbapenem-sensitive isolates (the ceftazidime and imipenem-sensitive strains) were selected to analyze the distribution of *bla*OXA genes and IS*Aba*1 element. As shown in Table 1, the current study revealed that IS*Aba*1 was found in 96 (96%) of the resistant isolates and in 40 (74%) of the sensitive isolates. In the carbapenem-resistant isolates, *bla*OXA-51-like and IS*Aba*1 were found in 28%, whereas *bla*OXA-23-like and IS*Aba*1 were found in 3% isolates. In the susceptible isolates, *bla*OXA-51-like and IS*Aba*1 were found in 3% of the isolates, and *bla*OXA-23-like and IS*Aba*1 were found in 1% of the susceptible isolates. This study demonstrated that *bla*OXA-51-like and IS*Aba*1 as well as *bla*OXA-23-like and IS*Aba*1 were found in both of the carbapenem-resistant and susceptible *A. baumannii* isolates (Table 1). *A. baumannii* strains that produce extended-spectrum beta-lactamases (ESBLs) are crucial nosocomial pathogens. Analysis of ESBL-producing strains commonly reveals *bla*NDM and *bla*PER-1 genes. In this study, *bla*NDM was not found in the test strains, but 38.3% of the test isolates carried *bla*PER-1, which might be responsible for the resistance to ESBLs and cephalosporins [16]. In addition, *bla*ADC and *tnp*A genes were found in 64.9% and 74% of the isolates, respectively. The PCR results showed that 68% of the isolates presenting a higher antibiotic resistance carried both *tnp*A and IS*Aba*1 genes (Table 1).

### 2.2. Detection of OXA Carbapenemases Expression

**Figure 2:**
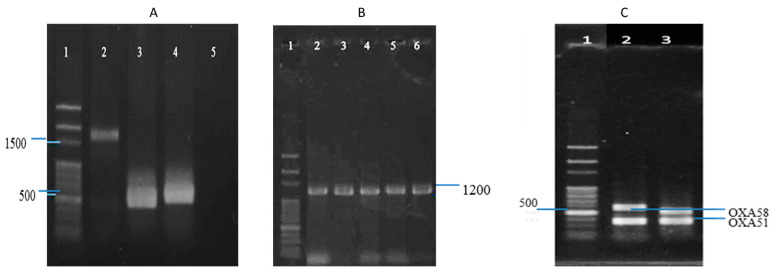
Detection of IS*Aba*1 element and genes encoding *OXA* carbapenemases (*bla*OXA-51-like, *bla*OXA-23-like and *bla*OXA-58-like) by multiplex PCR. (**A**): Lane 1, 100 bp DNA ladder; Lane 2, IS*Aba*1- *bla*OXA-23-like; Lane 3, IS*Aba*1; Lane 4, *bla*OXA-23-like; Lane 5, negative control. (**B**): Lane1, 100 bp DNA ladder; Lanes 2–6, IS*Aba*1-*bla*OXA-51-like. (**C**): Lane 1, 100 bp DNA ladder; Lane 2, *bla*OXA-58-like and *bla*OXA-51-like positive; Lane 3, *bla*OXA-51-like and *bla*OXA-23-like positive.

As shown in Figure 2, the multiplex PCR amplicons of IS*Aba*1- *bla*OXA-23-like and IS*Aba*1-*bla*OXA-51-like were about 1.5 kb and 1.2 kb in size, respectively (Figure 2A,B); the PCR mapping showed that IS*Aba*1 was located in the promoter region of the *bla*OXA-23-like and *bla*OXA-51-like genes. Multiplex PCR assay can detect and distinguish alleles encoding three subgroups of acquired OXA carbapenemases (*bla*OXA-23-like, *bla*OXA-51-like, and *bla*OXA-58-like). Detection results of carbapenemases encoding genes through multiplex PCR revealed that seven isolates harbored both *bla*OXA-51-like and *bla*OXA-23-like genes; three isolates carried both *bla*OXA-51-like and *bla*OXA-58-like genes; no isolate had both *bla*OXA-58-like and *bla*OXA-23-like genes; and the remaining isolates carried a single *bla*OXA gene (*bla*OXA-51-like, *bla*OXA-23-like, or *bla*OXA-58-like) (Figure 2C).

### 2.3. Repetitive Element PCR-Mediated DNA Fingerprinting (Rep-PCR)

**Figure 3:**
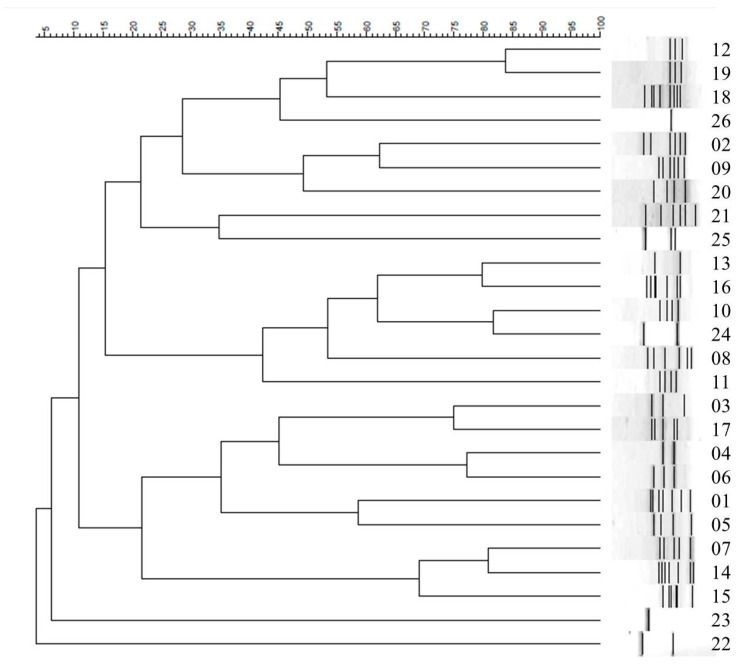
Rep-PCR–generated dendrogram for the 154 strains of *A. baumannii*.

The epidemiological typing of the 154 *A. baumannii* isolates was determined using repetitive element PCR-mediated DNA fingerprinting (Rep-PCR), and a total of 26 genotypes were identified. The amplicon size for ERIC-2 PCR was 200–3000 bp. Utilizing BioNumerics 6.6 (Applied Maths, Sint-Martens-Latem, Belgium), a clustering analysis was executed using the Dice similarity coefficient and the unweighted pair group method with arithmetic mean (UPGMA) algorithm. (Figure 3).

The most prevalent genotype was type 6 (n = 70; 45.4%). Among the 70 isolates in type 6, 38 (54.3%) showed strong biofilm formation; 94.2% and 96% of the isolates carried *bla*OXA-51-like and IS*Aba*1 genes, respectively. The second most prevalent genotype was type 4 (n = 20; 13%); approximately 55% (n = 11) of the isolates in this type were strong biofilm formers. Among type 4 isolates, 99% harbored both *bla*OXA-51-like and *bap* genes. Among the 26 genotypes, only the strains from type 13 did not carry *bla*OXA-51-like genes.

### 2.4. Correlation between the Antibiotic-Resistant Genes, Antibiotic Susceptibility, Minimum Inhibitory Concentration, Biofilm-Related Genes and Biofilm Formation

The distribution of genes, DISK (Disk Diffusion Method) and MIC (Minimum Inhibitory Concentration) results according to the high (23) and low (01) biofilm stress category was summarized in Supplementary Table S1. The gene *bap* showed a significantly different proportion in biofilm stress classification, especially higher in the high biofilm stress classification. The Tetracycline, Sulfamethoxazole-Triethoprim, Gentamicin, and Ticarcillin in DISK results showed significantly different proportions in biofilm stress classification. While in MIC results, only the Gentamicin showed a significantly different proportion in biofilm stress classification. The gene *bla*OXA-51-like showed a significantly higher proportion in the high biofilm stress category (high vs. low: 90.8% vs. 76.5%, *P* = 0.025). The Tetracycline (66.7% vs. 47.1%, *P* = 0.037), Sulfamethoxazole-Triethoprim (90.8% vs. 70.6%, *P* = 0.002), Gentamicin (67.5% vs. 47.1%, *P* = 0.029), Ceftazidime (77.5% vs. 58.8%, *P* = 0.030) and Ticarcillin (95.0% vs. 79.4%, *P* = 0.004) with resistance response in DISK results showed significantly higher proportion in high biofilm stress category. Similarly, only Gentamicin (73.7% vs. 29.4%, *P* = 0.001) with resistance response in MIC results showed a significantly higher proportion in the high biofilm stress category.

Based on the univariate logistic regression analysis, the correlation between biofilm stress category, gene, DISK and MIC (Minimum Inhibitory Concentration) are shown in Supplementary Table S2. The gene blaOXA-51-like showed a significantly higher odds ratio (OR = 3.05, 95% Confidence interval [CI] = 1.118.34, *P* = 0.030) associated with the high biofilm stress category. In DISK results, Tetracycline (OR = 0.44, 95% CI = 0.210.96, *P* = 0.040), Sulfamethoxazole-Triethoprim (OR = 0.24, 95% CI = 0.090.63, *P* = 0.004), Gentamicin (OR = 0.43, 95% CI = 0.20–0.93, *P* = 0.032), Ceftazidime (OR = 0.41, 95% CI = 0.19–0.93, *P* = 0.032) and Ticarcillin (OR = 0.20, 95% CI = 0.06–0.65, *P* = 0.007) with stimulate response showed lower odds to obtain higher biofilm stress compared to those with resistance response. In MIC results, Gentamicin (OR = 0.15, 95% CI = 0.04–0.49, *P* = 0.002) with stimulate response showed lower odds of obtaining higher biofilm stress compared to those with resistance response. While the stepwise selection multivariate logistic regression analysis showed only the Sulfamethoxazole-Triethoprim (OR = 0.28, 95% CI = 0.09–0.88, *P* = 0.030) in DISK results, Gentamicin (OR = 0.10, 95% CI = 0.02–0.42, *P* = 0.002) in MIC results with stimulate response have lower odds to obtain high biofilm stress, and Tetracycline (OR = 4.79, 95% CI = 1.04–22.04, *P* = 0.044) in MIC results with stimulate response have higher odds to obtain higher biofilm stress.

**Figure 4:**
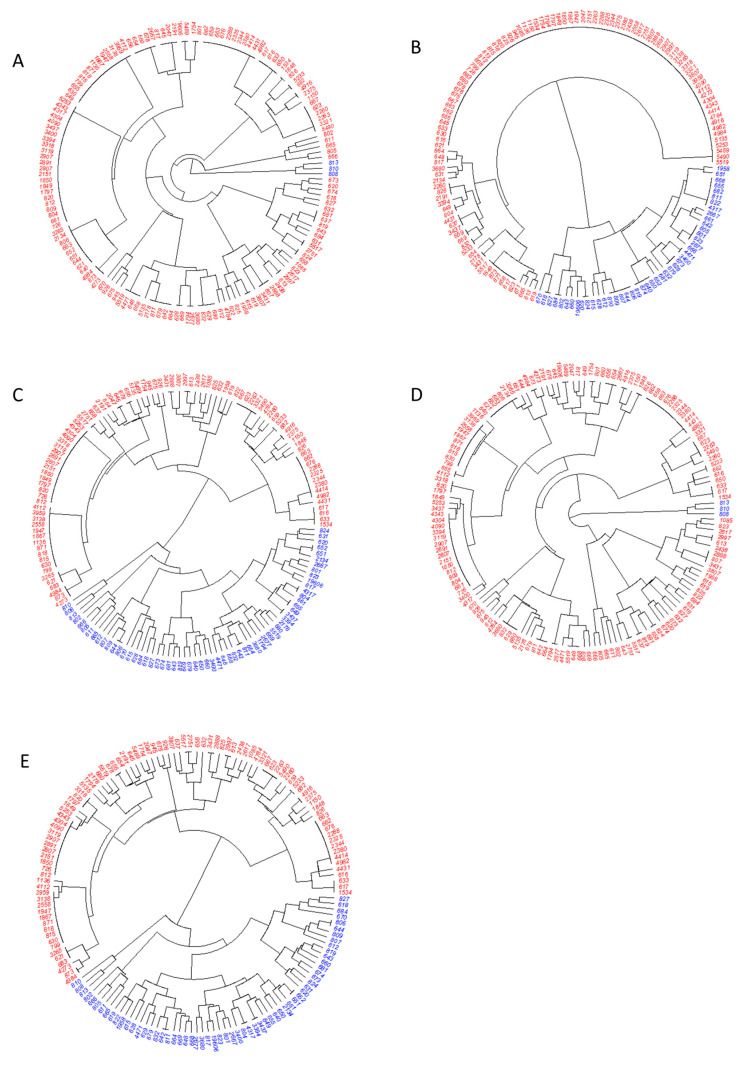
**Hierarchical clustering dendrogram.** Red font indicates cluster 1 and blue font indicates cluster 2. (**A**) Derived from clustering inclusion criteria with Gene results. (**B**) Derived from clustering inclusion criteria with DISK results. (**C**) Derived from clustering inclusion criteria with gene and DISK results. (**D**) Derived from clustering inclusion criteria with gene and MIC results. (**E**) Derived from clustering inclusion criteria with gene, DISK and MIC results.

**Figure 5:**
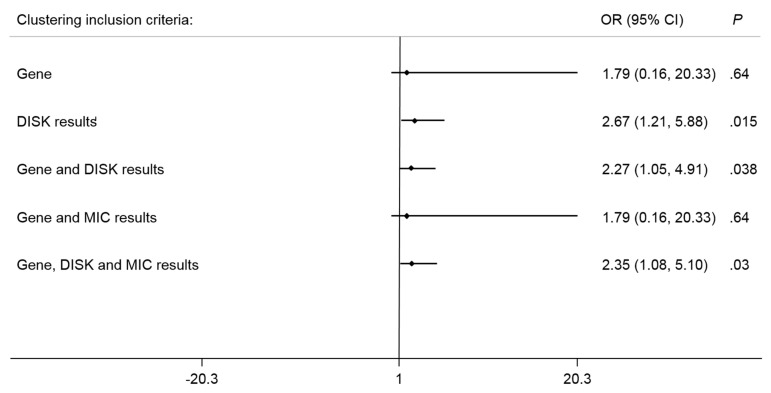
Forest plot showing associations of higher odds of obtaining high biofilm stress in clustering results with five clustering inclusion criteria.

The hierarchical clustering dendrograms derived from five clustering inclusion criteria (1) gene results, (2) DISK results, (3) gene and DISK results, (4) gene and MIC results, and (5) gene, DISK and MIC results were illustrated in Figure 4A–E, respectively. The distribution of samples in each cluster derived from hierarchical clustering results with five clustering inclusion criteria was summarized in Table 2. The significant higher difference in high and low biofilm stress was found in cluster 2 of DISK results (high vs. low: 75.0% vs. 52.9%, *P* = 0.013), gene and DISK results (64.2% vs. 44.1%, *P* = 0.035) and gene, DISK and MIC results (65.0% vs. 44.1%, *P* = 0.028). The performance examined using ROC analysis showed similar results, clustering inclusion criteria with DISK results (AUC = 0.610, 95% CI = 0.517–0.704) showed the highest AUC with acceptable dichotomous performance for the biofilm stress category. In addition, clustering inclusion criteria with both gene and DISK results (AUC = 0.600, 95% CI = 0.505–0.697) and gene, DISK and MIC results (AUC = 0.604, 95% CI = 0.509–0.699) also showed acceptable dichotomous performance for biofilm stress category. Figure 5 illustrates the forest plot which shows associations between clustering results with five clustering inclusion criteria and biofilm category. Similarly, consistently superior results were found in clustering inclusion criteria with DISK results (OR = 2.67, 95% CI = 1.21–5.88, *P* = 0.015), gene and DISK results (OR = 2.27, 95% CI = 1.05–4.91, *P* = 0.038) and gene, DISK and MIC results (OR = 2.35, 95% CI = 1.08–5.10, *P* = 0.030). Compare to cluster 1, all cluster 2 derived from the three clustering inclusion criteria were likely to gain higher odds of obtaining higher biofilm stress.

## 3. Discussion

The emergence and dissemination of multidrug-resistant (MDR) pathogens within healthcare environments have been driven by the excessive use of antimicrobials and the disregard for established hand hygiene protocols. Consequently, it is imperative to implement suitable measures to preempt nosocomial outbreaks [17,18]. *A. baumannii*, an increasingly common pathogen, is closely associated with hospital-acquired infections [19]. The proliferation of MDR *A. baumannii* strains poses an increasing threat in hospitals [20]. In our previous study, we examined the antibiotic susceptibility of *A. baumannii* strains against eleven antibiotics, including five antibiotic classes. The results revealed that most of the test isolates were MDR strains, and the risks associated with MDR strains were on the rise in the hospital in question [16].

Carbapenems are antibiotic agents commonly used for the treatment of multidrug resistance in *A. baumannii* strains [2]. However, carbapenem-resistant *A. baumannii* (CRAB) has become common worldwide [21]. Carbapenems belong to the β-lactam class of antibiotics, and lactam resistance is highly common in *A. baumannii* strains, particularly those isolated from hospitals [9,21]. Their distinctive molecular configuration arises from the inclusion of a carbapenem and beta-lactam ring, providing remarkable resilience against majority of beta-lactamases (enzymes that deactivate beta-lactams). This includes resistance to compounds such as ampicillin and carbenicillin, as well as extended-spectrum beta-lactams [22]. Additionally, carbapenem antibiotics are typically unaffected by emerging antibiotic resistance, including other beta-lactams used in hospitals as a last resort [23]. In Taiwan, the initial detection of CRAB occurred in the late 1990s, followed by a swift escalation noted in the late 2000s [24]. In our previous study, carbapenem antibiotic resistance was observed in 78.8% of the test isolates, indicating a higher proportion [16]. Primary contributors to the proliferation of multidrug resistance, including CRAB, involve inadequate compliance with infection control protocols and excessive utilization of specific antimicrobials.

Biofilms are collections of microorganisms enclosed within a matrix, functioning as collaborative consortia [25]. In our previous study, we investigated the relationships among antibiotic resistance, biofilm formation, and the related genes (*bap*, *csuE*, *omp*A and *bla*PER-1) in clinical isolates of *A. baumannii*. Based on statistical analysis, we observed that the *omp*A and *bap* genes exert an influence on both biofilm formation and antibiotic resistance patterns [16].

Rep-PCR is a useful molecular method for investigating genetic patterns of *A. baumannii* isolates [26]. In this study, Rep-PCR was used for genetic investigation, as ERIC-PCR is a reliable technique for discriminating intraspecific variations [27]. The phenotypicand molecular typing of *A. baumannii* isolates is essential for preventing outbreaks of MDR strains. The analysis of the correlation between Rep-PCR fingerprint and antibiotic susceptibility profiles revealed that all strains of genotype 13 exhibited aminoglycoside susceptibility in the disk diffusion and MIC tests. Additionally, genotype 6 was the most common type. These findings may have resulted from nosocomial infections attributed to cross-contamination, patient transfer, and environmental contamination, all of which play key roles in *A. baumannii* infection epidemics [26]. Statistical analyses revealed several strains belonging to genotype 6. These strains are not only classified under the same type in Rep-PCR but also categorized under biofilm formation classification and genotyping, indicating a correlation between biofilm formation classification and genotype.

Carbapenems are used as the final resort in treating infections caused by MDR bacteria, and carbapenem resistance within gram-negative organisms poses a significant global challenge. The rapid dissemination of carbapenemase-producing *Pseudomonas* spp. and *Acinetobacter* spp. has been witnessed on a worldwide scale in recent times [27]. Carbapenemases encompass Ambler class A β-lactamases (KPCs), class B metallo-β-lactamases (MBLs) such as VIMs, IPMs, and NDMs, and class D carbapenemhydrolyzing oxacillinases of (OXA-23, OXA-24, OXA-48, OXA-51, and OXA-58) [27].

Since the first report of *bla*OXA-51 [28], a high number of closely related variants have been found and collectively named “*bla*OXA-51-like” genes [29]. Numerous recent studies have reported that most *A. baumannii* isolates contain the *bla*OXA-51-like gene [30]. Furthermore, this gene is commonly identified in nosocomial isolates originating from both Asia and Europe, frequently coexisting with the *bla*OXA-23-like gene [31]. In the present study, up to 88% of the test strains harbored the *bla*OXA-51-like gene. Determining the precise role of *bla*OXA-51-like in carbapenem resistance within *A. baumannii* has proven challenging in real-world conditions. This difficulty arises because all isolates possess a *bla*OXA-51-like gene, yet not all isolates exhibit carbapenem resistance. The suggestion put forth is that the *bla*OXA-51-like genes are usually exhibit low expression levels and might confer resistance solely when their expression is enhanced by the insertion of a promoter within an IS*Aba*1 insertion sequence element upstream of the gene [32]. A multiplex PCR assay designed to identify genes encoding species-specific *bla*OXAs demonstrated strong consistency across different OXA subgroups. Recently, an MDR *A. baumannii* harboring *bla*OXA-23, *bla*OXA-51, *bla*PER-1, *bla*ADC, and *bla*OXA-58 was responsible for high rates of infections in patients. Moreover, *bla*OXA-23-like is the most commonly acquired determinant of carbapenem resistance in *A. baumannii* [33]. Insertion sequences are commonly found in connection with OXA β-lactamase genes [34]. For instance, carbapenem resistance attributed to genes like*bla*OXA-51-like and *bla*OXA-23-like often occurs when these genes are located alongside insertion elements [35]. Mobilization events of this nature are linked to IS*Aba*1, which encodes not just a transposase but also a promoter, resulting in an elevated expression of the OXA gene and consequently leading to carbapenem resistance [36].

In the present study, 73% of the test strains possessed the flanking sequence, *tnp*A gene, which encodes for transposase. Additionally, 87% of the *tnp*A-harboring strains carried the IS*Aba*1 gene, and 68% of the test isolates contained both genes. These findings suggest that the resistance genes were widely distributed in a single hospital. The IS*Aba*1 insertion sequence facilitates overexpression of various oxacillinase genes by providing a promoter and conferring carbapenem resistance. In the present study, up to 77.3% (119/154) of the strains possessed both IS*Aba*1 and *bla*OXA-51-like genes. The drug susceptibility test revealed numerous carbapenem-resistant strains, and an association was found between the *bla*OXA-51-like gene and IS*Aba*1 gene located upstream of the *bla*OXA gene. Similar results were reported in a recent study that described most *A. baumannii* isolates from a Taiwan hospital and determined that they contained an IS*Aba*1-activated *bla*OXA51-like gene [37]. Another study reported that the insertion sequence of IS*Aba*1 upstream of carbapenemase genes could enhance the expression of resistant genes [33]. In one study, 3% of carbapenem-resistant isolates obtained PCR products using IS*Aba*1F and *bla*OXA-23R primers, and 23% of the isolates obtained PCR products using IS*Aba*1F and *bla*OXA-51R primers. Among the various IS elements, IS*Aba*1 stands out due to its inclusion of a promoter that influences the expression ofantibiotic-resistant genes [33].

In the majority of gram-negative bacteria, the acquisition of an extended-spectrum β-lactamase is accountable for the emergence of resistance to third-generation cephalosporins [28]. Cephalosporin resistance in *A. baumannii* is rapidly increasing due to the overexpression of intrinsic *Amp*C cephalosporinase [38]. Based on phylogenetic analyses, the genes encoding these enzymes have a common ancestor and result from the overproduction of *Acinetobacter*-derived cephalosporinases (ADCs) [21]. Recently, a unified terminology, ADC with *Amp*Cs of *A. baumannii*, has been recommended for the cephalosporinase family [21]. It has been observed that the presence of an IS*Aba*1 sequence upstream of the chromosomal *bla*ADC genes in *A. baumannii* potentially results in heightened expression of ADC-type ß-lactamases [2].Therefore, carbapenemase and cephalosporinase genes, which are responsible for resistance to carbapenems and cephalosporins in *A. baumannii* clinical isolates, were analyzed. In the present study, although IS*Aba*1 was not detected upstream of the *bla*ADC gene, 44% of the strains simultaneously possessed *bla*ADC and *bla*OXA-51 genes, which are among the causes of the increase in drug resistance. The present study primarily conducted a four-way comparison among biofilm formation, biofilm-producing genes, drug-resistant genes, and drug susceptibility. Based on the statistical analyses, *bla*OXA-51-like is associated with greater biofilm formation and antibiotic resistance. The *bla*OXA51- like gene is a class D carbapenemase gene; these genes are associated with the recent emergence and transmission of MDR bacteria in hospitals. Therefore, the carbapenemase-like resistance gene could be associated with the formation of stronger biofilms and antibiotic resistance. Thus, in hospitals where there is a high prevalence of resistance to broad-spectrum cephalosporins, it is crucial to carefully select appropriate antibiotics. This can be achieved through the use of a combination of beta-lactam and beta-lactamase inhibitors, or carbapenems [33].

While the findings of this study establish a correlation between the phenotypes and relevant genotypes in drug-resistant bacteria, there are still several limitations that should be noted: (1). The bacterial strains were sourced solely from a single hospital, which might limit the comprehensiveness of diversity analysis. (2). Further investigation is required to explore more options for testing antibiotic-resistant genes, such as *bla*KPC, *bla*VIM, *bla*IMP, *bla*OXA-48, and others, in order to facilitate more profound comparative analyses. (3). Conducting sequencing comparisons of the PCR products of antibiotic-resistant genes may potentially reveal more subtle correlations, such as the diversity of single nucleotide polymorphism (SNP). Moreover, the significance of the statistical analysis results could potentially be enhanced by increasing the number of strain isolates in this study.

## 4. Materials and Methods

### 4.1. Bacterial Strains

A collection of 154 non-duplicate antibiotic-resistant strains of *A. baumannii* were obtained from patients’ blood or sputum at Ditmanson Medical Foundation Chia-Yi Christian Hospital (Chiayi, Taiwan). These strains were preserved at –80 ℃ and cultivated overnight at 37 ℃ on Mueller-Hinton agar (MHA). The reference strain utilized in this investigation was *Acinetobacter baumannii* ATCC19606, procured from the Food Industry Research and Development Institute (Hsinchu, Taiwan).

### 4.2. Molecular Characterization of Antimicrobial Resistance Determinants

The identification of all isolates was carried out through16S rRNA gene sequencing. To detect various Ambler class *bla* genes and mobile genetic elements, a series of polymerase chain reactions (PCR) were conducted. The identified *bla* gene groups encompassed the following classes: class A, specifically *bla*PER; class B, represented by *bla*NDM; class C, which included *bla*ADC; and class D, comprising five subgroups of OXA carbapenemase genes (namely, *bla*OXA-23-like, *bla*OXA-24-like, *bla*OXA-51-like, *bla*OXA-58-like and *bla*OXA-143-like) [39]. In addition, PCR analysis detected the IS element (IS*Aba*1) and the transposon element (*tnp*A) [40]. The primer sequences employed in this study are detailed in Table 3. All PCR assays were executed using PCR Red Master Mix (AMPLIQON, Paris) within an ABI thermocycler (Applied Biosystems 2720, Kenilworth, NJ, USA). For quality control, positive and negative controls were included in all PCR assays. The amplification reactions were set up in a total volume of 25 µL, including 5 ng of genomic DNA, 2.0 U of *Taq* DNA polymerase, 10 mM dNTP mix at a final concentration of 0.2 mM, 50 mM MgCl_2_ at a final concentration of 1.5 mM, 1 mM of each primer, and PCR buffer. The PCR conditions included an initial denaturation step at 94 ℃ for 5 min, followed by 35 cycles of denaturation step at 94 ℃ for 60 s, an annealing temperature specific to each gene (as per Table 3) for 1 min, an extension step at 72 ℃ for 45 s, and a final extension at 72 ℃ for 5 min. Subsequently, the PCR products were subjected to gel electrophoresis on a 1% agarose gel at 100 V. The gel was stained with ethidium bromide solution and the results were visualized using a UV gel documentation system. A multiplex PCR approach was employed to simultaneously amplify four carbapenem resistance genes (oxacillinases), including *bla*OXA-51-like (which also served for the identification of isolates at the species level), *bla*OXA-23-like, *bla*OXA-24-like, and *bla*OXA-58-like. The specific primer sets used for this purpose are outlined in Table 3.

### 4.3. Repetitive Element PCR-Mediated DNA Fingerprinting (Rep-PCR)

Genomic DNA served as the template for Rep-PCR, with ERIC-2 used as the primer [33]. The amplification conditions followed previously described protocols, albeit with some adjustments as outlined below: an initial denaturation at 94 ℃ for 5 min; followed by 30 cycles consisting of denaturation at 94 ℃ for 1 min, annealing at 45 ℃ for 1 min, extension at 72 ℃ for 2 min, and a final extension at 72 ℃ for 16 min. The resulting amplification products were subjected to electrophoresis on a 1.0% agarose gel (Thermo), followed by staining with ethidium bromide. Visualization of the products was accomplished using a UV gel documentation system.

### 4.4. Data Analyses

The study summarized the distribution of gene, DISK (disk diffusion method), and MIC (minimum inhibition concentration) results based on two categories of biofilm stress. The low biofilm stress category consisted of samples with biofilm stress levels of 0 and 1, while the high biofilm stress category included samples with biofilm stress levels of 2 and 3. The differences in gene, DISK, and MIC results between the two biofilm stress categories were assessed using either chi-squared test or Fisher’s exact test. Univariate logistic regression was employed to examine the association of each gene, DISK, and MIC results with high biofilm stress. The stepwise selection model with inclusion criteria *p*-value of 0.2 is built to check the significant covariance for high biofilm stress.

The distribution of each cluster derived from five inclusion criteria through hierarchical clustering was analyzed using frequency and percentage. The performance of each clustering inclusion criteria based on the biofilm stress category was evaluated by calculating the area under the receiver operating characteristic curve (AUC). Logistic regression analysis was conducted to determine the odds ratios (OR) of high biofilm stress between dichotomous clusters in each clustering inclusion criteria. The outcomes were displayed in a forest plot, with statistical significance defined as a *p*-value below 0.05. All statistical computations were conducted utilizing Stata version 14.0 (StataCorp. 2015. Stata Statistical Software: Release 14. College Station, TX: StataCorp LP, College Station, TX, USA) [41].

## 5. Conclusions

The study delved into the molecular genotypes and phenotypes of clinical antibiotic-resistant *A. baumannii*. It aimed to uncover the interrelationships between antibiotic-resistance, antibiotic resistant genes, biofilm formation, and genes associated with biofilm development. According to the detected biofilm-related genes, namely *bap*, *csu*E, *bla*PER-1 and *omp*A genes, the *bap* and *omp*A genes were the most frequently present in the clinical isolates [16]. In Rep-PCR genotyping, we observed that 45% of the isolates belonged to genotype 6 and 97% of the strains in genotype 6 harbored the *omp*A gene, whereas 90% harbored the *bap* gene. Among the genotype 6 strains, 90% were strong biofilm producers, 94% had the *bla*OXA-51-like gene, and 96% carried the IS*Aba*1 gene. IS*Aba*1, the most common IS located upstream of *bla*OXA-51-like and *bla*OXA-23-like, could be responsible for carbapenem resistance. The findings indicated that IS*Aba*1 functions as a promoter for both *bla*OXA-51-like and *bla*OXA-23-like genes. Our investigation highlighted the potential impact of genes *bap*, *omp*A, IS*Aba*1, and *bla*OXA-51-like on both biofilm formation and antibiotic resistance patterns. These conclusions were supported by the statistical outcomes of hierarchical clustering dendrograms. These mechanisms provide valuable insights into comprehending the intricate connection between biofilm production and antibiotic resistance in *A. baumannii*, as well as shedding light on the potential routes of transmission for clinical isolates.

## Figures and Tables

**Table 1:** Distribution of carbapenemase genes, IS*Aba*1 and *tnp*A elements in *A. baumannii* isolates.

Genes	Number of Isolates/% *	Carbapenems Resistant Isolates ^+^ Number of Isolates/%	Carbapenems Sensitive Isolates ^#^ Number of Isolates/%
*bla*_OXA-51-like_	135/87.6	89/89	46/85
*bla*_OXA-23-like_	7/4.5	5/5	2/3
*bla*_OXA-58-like_	5/3.2	-	-
*bla*_OXA-24-like_	0/0	-	-
*bla*_OXA-143-like_	0/0	-	-
*tnp*A	114/74	-	-
IS*Aba*1	136/88.3	96/96	40/74
*bla*_NDM_	0/0	-	-
*bla*_ADC_	100/64.9	-	-
*bla*PER-1	59/38.3	-	-
IS*Aba*1 + *bla*_OXA-23-like_	4/2.6	3/3	1/1
IS*Aba*1 + *bla*_OXA-51-like_	31/19.4	28/28	2/3
*bla*_OXA-51-like_ + *bla*_OXA-23-like_	7/4.5	-	-
*bla*_OXA-51-like_ + *bla*_OXA-58-like_	3/1.9	-	-
*bla_OXA-58-like_ + bla_OXA-23-like_*	0/0	-	-
IS*Aba*1 + *tnp*A	105/68	-	-

* Total isolates (n = 154). ^+^ The Carbapenems resistant isolates were selected from the ceftazidime and imipenem-resistant strains, total isolates (n = 100). ^#^ The Carbapenems sensitive isolates were selected from the ceftazidime and imipenem sensitive strains, total isolates (n = 54). - undetected.

**Table 2:** The distribution of isolates in each cluster derived from hierarchical clustering results with five clustering inclusion criteria.

Clustering Inclusion Criteria *	Total	Low Biofilm Formation (0–1)		High Biofilm Formation (23)		*P*	*AUC * (95%* CI*)
		n	%	n	%		
(1) Gene results						0.530	
Cluster 1	3	1	2.9%	2	1.7%		0.506
Cluster 2	151	33	97.1%	118	98.3%		(0.4750.537)
(2) DISK results						**0.013**	
Cluster 1	46	16	47.1%	30	25.0%		0.610
Cluster 2	108	18	52.9%	90	75.0%		(0.5170.704)
(3) Gene and DISK results						**0.035**	
Cluster 1	62	19	55.9%	43	35.8%		0.600
Cluster 2	92	15	44.1%	77	64.2%		(0.5050.697)
(4) Gene and MIC results						0.530	
Cluster 1	3	1	2.9%	2	1.7%		0.506
Cluster 2	151	33	97.1%	118	98.3%		(0.4750.537)
(5) Gene, DISK and MIC results						**0.028**	
Cluster 1	61	19	55.9%	42	35.0%		0.604
Cluster 2	93	15	44.1%	78	65.0%		(0.509–0.699)

*p*-value is estimated using chi-squared or Fisher’s exact test. AUC: Area Under Curve. * (1) Gene results, (2) DISK results, (3) Gene and DISK results, (4) Gene and MIC results, and (5) Gene, DISK and MIC results

**Table 3:** Primers used for gene detection in this study.

Primers	Primer Sequence (5′-3′)	Product Size (bp)	Target	References
*bla*_OXA-51-like_	TAATGCTTTGATCGGCCTTGTG GATTGCACTTCATCTTGG	353	*OXA* carbapenemase genes	[39]
*bla*_OXA-23-like_	GATCGGATTGGAGAACCAGA ATTTCTGACCGCATTTCCAT	501	*OXA* carbapenemase genes	[39]
*bla*_OXA-58-like_	AAGTATTGGGGCTTGTGCT GCCCCTCTGCGCTCTACATAC	599	*OXA* carbapenemase genes	[39]
*bla*_OXA-24-like_	GGTTAGTTGGCCCCCTTAAA AGTTGAGCGAAAAGGGGATT	249	*OXA* carbapenemase genes	[22]
*bla*_OXA-143-like_	TGGCACTTTCAGCAGTTCCT TAATCTTGAGGGGGCCAACC	149	*OXA* carbapenemase genes	[33]
*tnp*A	ATGCAGCGCTTCTTTGCC AGG AATGAT TGG TGACAATGA AG	354	*tnp*A gene	[38]
ERIC-2	AAGTAAGTGACTGGGGTGAGCG	random	Rep-PCR	[38]
IS*Aba*1	CATTGGCATTAAACTGAGGAGAAA TTGGAAATGGGGAAAACGAA	451	IS*Aba*1	[33]
*bla*_PER-1_	GCAACTGCTGCAATACTCGG ATGTGCGACCACAGTACCAG	900	*bla*_PER_	[40]
*bla*_NDM_	GGTTTGGCGATCTGGTTTTC CGGAATGGCTCATCACGA TC	621	*bla*_NDM_	[40]
*bla*_ADC_	TAA ACACCACATATGTTCCG ACT TACTTCAACTCGCGACG	663	*bla*_ADC_	[21]

## Data Availability

Not applicable.

## Supplementary Materials

The following supporting information can be downloaded at: https://www.mdpi.com/article/10.3390/antibiotics12091357/s1, Table S1: Distribution of gene, DISK and MIC results in high and low biofilm stress categories. (n = 154); Table S2: Logistic regression analysis for association between biofilm stress category and gene, DISK and MIC results.
