# Supplementary material for: Prevalence of Carbapenem Resistance Genes among Acinetobacter baumannii Isolated from a Teaching Hospital in Taiwan
Source: Antibiotics (Basel). 2023 Aug 23;12(9):1357. doi: 10.3390/antibiotics12091357 (PMC10525170; doi:10.3390/antibiotics12091357)
Supplement: Supplementary file 1 [file antibiotics-12-01357-s001.zip › antibiotics-2506831-supplementary.docx]

Supplementary Table 1. Distribution of gene, DISK and MIC results in high and low biofilm stress categories. (n=154)

| Variables | Low biofilm stress (0-1) | |  | High biofilm stress (2-3) | | *P* |
| --- | --- | --- | --- | --- | --- | --- |
|  | n | % |  | n | % |  |
| Total cases, row % | 34 | 22.1% |  | 120 | 77.9% | *-* |
| Gene |  |  |  |  |  |  |
| *OXA51* | 26 | 76.5% |  | 109 | 90.8% | **0.025** |
| *OXA23* | 1 | 2.9% |  | 6 | 5.0% | 0.518 |
| *OXA58* | 2 | 5.9% |  | 3 | 2.5% | 0.305 |
| *bap* | 23 | 67.6% |  | 99 | 82.5% | 0.060 |
| *bla PER* | 9 | 26.5% |  | 50 | 41.7% | 0.108 |
| *tnpA gene* | 22 | 64.7% |  | 91 | 75.8% | 0.195 |
| *ISAbal* | 27 | 79.4% |  | 107 | 89.2% | 0.135 |
| *ISAba1F/OXA23R* | 1 | 2.9% |  | 2 | 1.7% | 0.530 |
| *ISAba1F/OXA51R* | 4 | 11.8% |  | 27 | 22.5% | 0.126 |
| *blaADC* | 23 | 67.6% |  | 77 | 64.2% | 0.707 |
| DISK |  |  |  |  |  |  |
| Tetracycline |  |  |  |  |  | **0.037** |
| R | 16 | 47.1% |  | 80 | 66.7% |  |
| S | 18 | 52.9% |  | 40 | 33.3% |  |
| Sulfamethoxazole-Triethoprim |  |  |  |  |  | **0.002** |
| R | 24 | 70.6% |  | 109 | 90.8% |  |
| S | 10 | 29.4% |  | 11 | 9.2% |  |
| Gentamicin |  |  |  |  |  | **0.029** |
| R | 16 | 47.1% |  | 81 | 67.5% |  |
| S | 18 | 52.9% |  | 39 | 32.5% |  |
| Ceftazidime |  |  |  |  |  | **0.030** |
| R | 20 | 58.8% |  | 93 | 77.5% |  |
| S | 14 | 41.2% |  | 27 | 22.5% |  |
| Ticarcillin |  |  |  |  |  | **0.004** |
| R | 27 | 79.4% |  | 114 | 95.0% |  |
| S | 7 | 20.6% |  | 6 | 5.0% |  |
| MIC (n=74) |  |  |  |  |  |  |
| Total cases, row % | 17 | 23.0% |  | 57 | 77.0% |  |
| Tetracycline |  |  |  |  |  | 0.493 |
| R | 7 | 41.2% |  | 23 | 40.4% |  |
| S | 10 | 58.8% |  | 34 | 59.6% |  |
| Sulfamethoxazole-Triethoprim |  |  |  |  |  | 0.105 |
| R | 9 | 52.9% |  | 42 | 73.7% |  |
| S | 8 | 47.1% |  | 15 | 26.3% |  |
| Gentamicin |  |  |  |  |  | **0.001** |
| R | 5 | 29.4% |  | 42 | 73.7% |  |
| S | 12 | 70.6% |  | 15 | 26.3% |  |
| Ceftazidime |  |  |  |  |  | 0.567 |
| R | 11 | 64.7% |  | 41 | 71.9% |  |
| S | 6 | 35.3% |  | 16 | 28.1% |  |
| Ticarcillin |  |  |  |  |  | 0.253 |
| R | 13 | 76.5% |  | 50 | 87.7% |  |
| S | 4 | 23.5% |  | 7 | 12.3% |  |

S: stimulate; R: resistance.

*P*-value is estimated using chi-squared test or Fisher’s exact test.

Supplementary Table 2. Logistic regression analysis for association between biofilm stress category and gene, DISK and MIC results.

| Variables | Univariate | | |  | Stepwise (*p* < 0.2) Model | | |
| --- | --- | --- | --- | --- | --- | --- | --- |
|  | OR | 95% CI | *P* |  | OR | 95% CI | *P* |
| Gene |  |  |  |  |  |  |  |
| *OXA51* | 3.05 | 1.11 - 8.34 | **0.030** |  | 2.90 | 0.96 - 8.81 | 0.060 |
| *OXA23* | 1.74 | 0.2 - 14.94 | 0.615 |  | - | - | - |
| *OXA58* | 0.41 | 0.07 - 2.56 | 0.340 |  | 0.15 | 0.02 - 1.38 | 0.095 |
| *bap* | 2.25 | 0.95 - 5.32 | 0.064 |  | - | - | - |
| *bla PER* | 1.98 | 0.85 - 4.61 | 0.112 |  | 1.96 | 0.72 - 5.32 | 0.189 |
| *tnpA gene* | 1.71 | 0.76 - 3.88 | 0.198 |  | - | - | - |
| *ISAbal* | 2.13 | 0.78 - 5.87 | 0.142 |  | - | - | - |
| *ISAba1F/OXA23R* | 0.56 | 0.05 - 6.36 | 0.640 |  | - | - | - |
| *ISAba1F/OXA51R* | 2.18 | 0.70 - 6.73 | 0.176 |  | - | - | - |
| *blaADC* | 0.86 | 0.38 - 1.92 | 0.707 |  | - | - | - |
| DISK |  |  |  |  |  |  |  |
| Tetracycline (S vs R) | 0.44 | 0.21 - 0.96 | **0.040** |  | - | - | - |
| Sulfamethoxazole-Triethoprim (S vs R) | 0.24 | 0.09 - 0.63 | **0.004** |  | 0.28 | 0.09 - 0.88 | **0.030** |
| Gentamicin (S vs R) | 0.43 | 0.20 - 0.93 | **0.032** |  | - | - | - |
| Ceftazidime (S vs R) | 0.41 | 0.19 - 0.93 | **0.032** |  | - | - | - |
| Ticarcillin (S vs R) | 0.20 | 0.06 - 0.65 | **0.007** |  | - | - | - |
| MIC |  |  |  |  |  |  |  |
| Tetracycline (S vs R) | 1.03 | 0.34 - 3.11 | 0.951 |  | 4.79 | 1.04 - 22.04 | **0.044** |
| Sulfamethoxazole-Triethoprim (S vs R) | 0.40 | 0.13 - 1.23 | 0.111 |  | - | - | - |
| Gentamicin (S vs R) | 0.15 | 0.04 - 0.49 | **0.002** |  | 0.10 | 0.02 - 0.42 | **0.002** |
| Ceftazidime (S vs R) | 0.72 | 0.23 - 2.26 | 0.568 |  | - | - | - |
| Ticarcillin (S vs R) | 0.45 | 0.12 - 1.79 | 0.261 |  | - | - | - |

S: stimulate; R: resistance.

*P*-value is estimated using chi-squared test or Fisher’s exact test.
